# Citral in lemon myrtle, lemongrass, litsea, and melissa essential oils suppress the growth and invasion of breast cancer cells

**DOI:** 10.1186/s12906-024-04511-4

**Published:** 2024-06-03

**Authors:** Takuya Nagata, Tadaaki Satou, Shinichiro Hayashi, Prabodh Satyal, Manabu Watanabe, Brannick Riggs, Yoshihisa Saida

**Affiliations:** 1 Department of Surgery, Toho University Ohashi Medical Center, Tokyo, Japan; 2 Department of Narita Pharmaceutical Sciences, International University of Health and Welfare, Chiba, Japan; 3 Department of Pharmacognosy, Faculty of Pharmaceutical Sciences, Toho University, Chiba, Japan; 4 Aromatic Plant Research Center, Lehi, UT USA; 5 Prime Meridian Healthcare, Pleasant Grove, Utah, USA

**Keywords:** Lemon myrtle, Lemongrass, Litsea, Melissa, Citral, Breast cancer

## Abstract

**Objective:**

Although cancer therapy suppresses recurrence and prolongs life, it may be accompanied by strong side effects; thus, there is a strong demand for the development effective treatments with fewer side effects. Cancer therapy using plant-derived essential oils is attracting attention as one promising method. This study investigated the antitumor effects of essential oil volatiles on breast cancer cells and identifies four essential oils that display antitumor activity.

**Methods:**

Breast cancer cells were cultured in a 96-well plate, then one of twenty essential oils was added dropwise to the central well. The plate was incubated at 37 °C for 48 h and the effect of the volatile components of each essential oil on the surrounding breast cancer cell growth ability was examined using an MTT assay. Gas chromatography was used to investigate the concentration of the transpiration components that may affect cancer cells.

**Results:**

Of the 20 essential oils, Lemongrass, Lemon myrtle, Litsea, and Melissa displayed strong anti-tumor effects. These essential oils inhibited the growth of nearby breast cancer cells, even when diluted more than 500-fold. The transpiration component of lemon Myrtle showed the strongest antitumor effect, but was the least cytotoxic to mononuclear cells in normal peripheral blood (PBMC). Each of these essential oils contained a very large amount of citral. The IC_50_ against breast cancer cells when citral was volatilized from each essential oil was 1.67 µL/mL for geranial and 1.31 µL/mL for neral. Volatilized citral alone showed strong anti-proliferation and infiltration-inhibiting effects.

**Conclusion:**

The transpiration components of Lemongrass, Lemon myrtle, Litsea, and Melissa are thought to inhibit breast cancer cell proliferation due to their high levels of citral.

## Introduction

Cancer is the leading cause of death in Japan, with one in two people developing cancer during their lifetime; breast cancer is the most common cancer among Japanese women, affecting one in nine women, and the age of breast cancer onset is relatively young and ranges from 40 to 65 years, with many mothers developing breast cancer before their children reach adulthood [1]. Various treatments exist for breast cancer, such as surgery, hormonal therapy, anticancer drugs, molecular-targeted drugs, radiation therapy, genomic diagnosis, and immune checkpoint inhibitors. Advances in these therapeutic methods and the development of new drugs have extended the survival prognosis of breast cancer patients [2]. In addition, genomic diagnosis has made it possible to select and administer highly effective anticancer drugs and molecularly targeted drugs; however, anticancer agents have various side effects, such as hair loss, malaise, nausea, diarrhea, constipation, cardiovascular effects, and febrile neutropenia [3]. Thus, anticancer drug treatments may significantly reduce the quality of life of breast cancer patients by imposing greater pain and stress. Since many breast cancer patients are not satisfied with current anticancer drug treatments, there is a strong demand for the development of new treatment methods with higher therapeutic effects and fewer side effects.

Essential oils are volatile compounds extracted from plants that have been found to have a variety of positive effects, such as antibacterial, antiviral, and stress-relieving effects. The global COVID-19 pandemic of recent years has caused an increase in the use of essential oils in an effort to relieve the negative effects of isolation and to help avoid contacting the virus. Commercially available essential oils are sprayed into rooms using diffusers, added to food as flavorings, used as bath additives, mixed with carrier oils for massage, and are generally widely used as a raw material for pharmaceuticals, foods, perfumes, and cosmetics [4, 5].

Terpenes, the main components of essential oils, are composed of two or more isoprene units (C5) and include monoterpenes (C10), sesquiterpenes (C15), diterpenes (C20), and terpenes with higher C25. Terpene derivatives with functional groups, such as carbonyl and hydroxy groups, are called terpenoids. The monoterpenoid aldehydes neral and geranial are cis–trans isomers, collectively referred to as citral, and are reported to have a wide range of biological properties (e.g., antitumor, anti-inflammatory, antibacterial, anti-fungal, antiviral, analgesic and anti-oxidative activities) [6, 7].

Globally, cancer prevalence is increasing yearly, which has caused the diagnosis and treatment of cancer to become very important issues. Synthetic anticancer compounds represent the largest segment of the current cancer treatment market, yet more than two-thirds of the drugs currently in clinical use are either derived directly from natural products or their biological activities are derived from natural products [8]. Many of the terpenes and terpenoids found in essential oils display antitumor activities [9]. Among these factors, detailed research into the antitumor effects of monoterpenes and sesquiterpenes, which have low molecular weights and high volatility, is expected to lead to the development of a new drug discovery field encompassing cancer treatment using scents.

In this study, we investigated the antitumor effects of the volatile components of twenty commercially available essential oils that are considered beneficial in the reduction of unpleasant symptoms and health maintenance. Of these, we selected the essential oils with the strongest antitumor effects for additional investigation, including the antitumor factors contained in the oils. The purpose of this research was to clarify the antitumor effects found for the volatile components of essential oils and to determine which constituent(s) in the tested component may be responsible for the antitumor and treatment-enhancing effects.

## Materials and methods

### Essential oil and adjustment

All essential oils, except for citral, were purchased from doTERRA (Preasant Grove Utah, USA) (Table 1). These essential oils were most often extracted using a steam distillation process and their constituents were evaluated using GC–MS. Citral was purchased from WAKO (Osaka, Japan), then dissolved in dimethyl sulfoxide (DMSO) to a concentration of 10^–2^ g/mL and stored at − 20 °C.

**Table 1:** The common and the scientific name of each essential oils

Common name	Scientific name
Cedar Wood	*Cedrus deodara, Juniperus virginiana*
Clove	*Eugenia caryophyllata*
Copaiba	*Copaifera spp.*
Frankincense	*Boswellia carteii, Boswellia Essential Oil*
Grapefruit	*Citrus* x *paradisi*
Hinoki	*Chamaecyparis obtusa*
Lavender	*Lavandula angustifolia*
Lemon	*Citrus limon*
Lemongrass	*Cymbopogon citratus, Cymbopogon flexuosus*
Lemon Myrtle	*Backhousia citriodora*
Lime	*Citrus aurantiifolia*
Litsea	*Litsea cubena*
Melissa	*Melissa officialis*
Niaouli	*Melaleuca viridiflora*
Wild Orange	*Citrus sinensis*
Oregano	*Origanum vulgare*
Tangerine	*Citrus reticulata*
Tea Tree	*Melaleuca alternifolia*
Thyme	*Thymus vulgaris*

### Cell lines and culture conditions

Two breast cancer cell lines (SKBR3 & MCF7) were purchased from the American Type Culture Collection (Manassas, VA, USA). Both breast cancer cells were cultured in Dulbecco’s modified Eagle medium (DMEM) (Sigma-Aldrich, St. Louis, MO, USA) containing 5% fetal calf serum (GIBCO, Gaithersburg, USA) and 1% Antibiotic–Antimycotic (Streptomycin-Amphotericin B and Penicillin) (Thermo Fisher Scientific, Tokyo, Japan) were added to all cultures. As controls, 3 mL peripheral blood samples were obtained from a healthy volunteer who was recruited before this study and provided informed consent. Venous blood was collected in heparinized tubes. Samples were processed and mononuclear cells (PBMC) were isolated using Lymphoprep (StemCell Technologies, Vancouver, Canada) centrifugation (800 × *g*, 20 min), and cultured in OpTmizer medium (Life Technologies Japan, Tokyo, Japan).

### Cell proliferation assay

The antitumor effects of the different essential oil components were quantified using a colorimetric cell viability assay (MTT) (3-(4,5-dimethylthiazol-2-yl)-2,5-diphenyltetrazolium bromide). First, the cancer cells were prepared in 96-well plates at a ratio of 1 × 10^4^ per 100 ul. Next, the essential oil components were administered to the cultured cells at concentrations of 0.098–50 µg/mL and incubated at 37 °C for 48 h. Afterwards, 10 µL of Solution I from the MTT cell proliferation assay kit (Roche Diagnostics, Tokyo, Japan) was added to each well. After reacting at 37 °C for 4 h, 100 µL of visualization solution was added to each well and allowed to react overnight at 37 °C. The absorbance at 570 nm was then measured using a plate reader (iMark plate reader; Bio Rad, Hercules, Calif.). The concentration resulting in 50% inhibition (IC_50_) was determined by curve fitting and was used as criterion to judge the cytotoxicity of a compound. For the transpiration component experiment, 50 µL of undiluted or 500-fold diluted essential oil were administered to the two central wells, and then allowed to react at 37 °C for 48 h. The MTT assay was performed to evaluate the antitumor activity of the volatile components of each essential oils.

### Morphology observations and the assessment of apoptosis response

The antitumor effects of essential oil components or essential oil transpiration components on breast cancer cell lines was evaluated by observing individual cells using a fluorescence microscope. Any breast cancer cells that reacted to the essential oils were double-stained with calcein-acetoxymethyl (AM) and propidium iodide (PI) from the cell stain double-staining kit (Dojindo Laboratories, Tokyo, Japan). This process stained the live cells green and the nuclei of the dead cells red. The stained breast cancer cells were observed under a fluorescence microscope (Olympus CKX-53, Tokyo, Japan) and any changes were photographed (Olympus DP28, Tokyo, Japan).

### Analysis of the concentration of volatile components

A total of 0.1 mL DMEM was administered to each well of a 96-well plate and MonoTrap RCC18 (GL Sciences, Tokyo, Japan) was added at regular intervals (proximal: 1.0 cm, middle: 2.0 cm, and distal: 3.0 cm) from the center. In addition, 50 µL of essential oil diluted to 0.02 mg/mL was administered to two wells in the center of the plate. Each plate was allowed to stand at 37 °C for 48 h. After the incubation, MonoTrap was moved in a glass container containing 2 mL hexane and subsequently sealed. The concentration of citral (geranial and neral, the two geometric isomers that are collectively termed citral) in each hexane solution was measured at the International University of Health and Welfare, using GC–MS-QP2010 (Shimadzu Corporation, Kyoto, Japan). A DB-5MS capillary column (30 m x 0.25 mm I.D.; 0.25 um non-polar column; Agilent Technologies, Tokyo, Japan) was used for analysis. Helium (99.99995%; 1.82 mL/min) was used as the carrier gas. The inlet line and source temperatures were set at 250 °C. The column oven temperatures were set at 40 °C for 2 min, then increased to 200 °C at a rate of 5 °C/min and maintained at 200 °C for 2 min.

Component analysis was performed on each essential oil using GC–MS at the Aromatic Plant Research Center (Lehi, Utah, USA). Components that were found in the essential oil at 0.1% or more were studied further.

### Cell migration and cell invasion assays

The cell migration and invasion assays were performed using the CytoSelect 24-well cell migration and invasion assay (8um, cololymetric format) kits (CELL BIOLABS, INC. San Diego, CA, USA). In the cell migration assay, SKBR3 breast cancer cells were plated in serum-free DMEM in the upper chamber at 1 × 10^5^ cells per well, respectively. The lower well contained DMEM with 10% FBS. The SKBR3 cells were allowed to migrate for 24 h. Then, the medium was aspirated from the inside of the insert, and the interior of the inserts was swabbed to remove non-migratory cells. The insert was stained with the cell stain solution for 10 min at room temperature, and the migratory cells were counted with a light microscope. Then, 200 µL of extraction solution was added to the insert and incubated for 10 min; after the incubation, 100 µL of sample was transferred to a 96-well microtiter plate and the OD at 560 nm was measured in the plate reader. The cell invasion assay was performed in a similar manner to the cell migration assay, except that the membrane filter was precoated with a uniform layer of dried basement membrane matrix solution. This basement membrane layer serves as a barrier to discriminate invasive cells from non-invasive cells.

### Statistical analysis

All data are expressed as means + / − standard deviation. One-way analysis of variance and post hoc Bonferroni’s multiple comparisons tests were used. The significance level was set at 5%. All statistical analyses were performed using Graph Pad prism version 9.4.1 (GraphPad software Inc., San Diego, Calif.).

## Results

### Antitumor effect by each essential oil transpiration component

The common and the scientific name of each essential oils that were analyzed in this study are listed in Table 1.

**Fig. 1:**
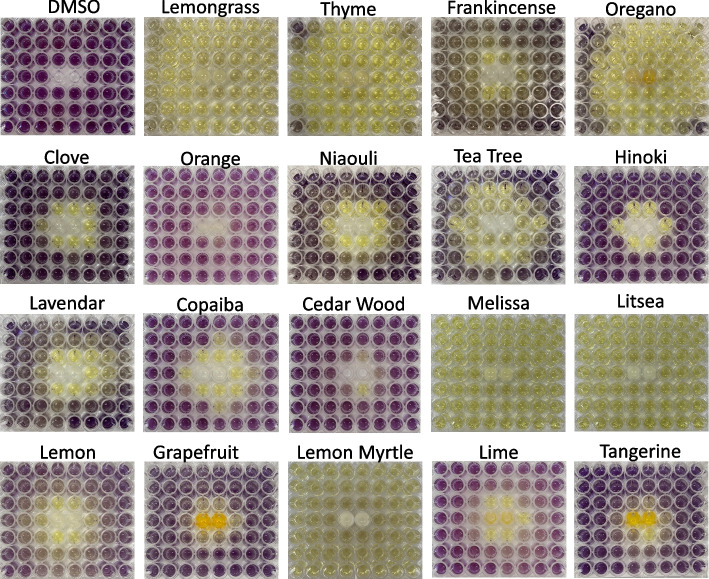
Antitumor effect of each essential oil transpiration component. Two types of breast cancer cells (SKBR3 and MCF7) were incubated in 96-well plates, and each essential oil was transferred into the two center wells. After 48 h, the MTT assay was performed, and the results are of the plate are shown

Figure 1 provides the results of the MTT assay, which shows the cancer cells that stopped proliferating in the yellow-stained areas and those that were actively proliferating in the navy-stained areas. DMSO was used as a control and did not change the proliferative ability of the breast cancer cells. Four essential oils stained the entire plate yellow: Lemongrass, Lemon myrtle, Litsea, and Melissa. Thyme and oregano essential oils displayed fairly strong tumor growth inhibitory effects. The citrus essential oils, such as lemon, orange, grapefruit, lime, and tangerine inhibited cancer cell growth only in the wells located near these essential oils. Frankincense, clove, niaouli, tea tree, hinoki, lavender, copaiba, and cedar wood essential oils also only inhibited proliferation in the nearby cancer cells. Based on these results, lemongrass, lemon myrtle, litsea and melissa were selected for further study.

### Antitumor effect of each essential oil

**Fig. 2:**
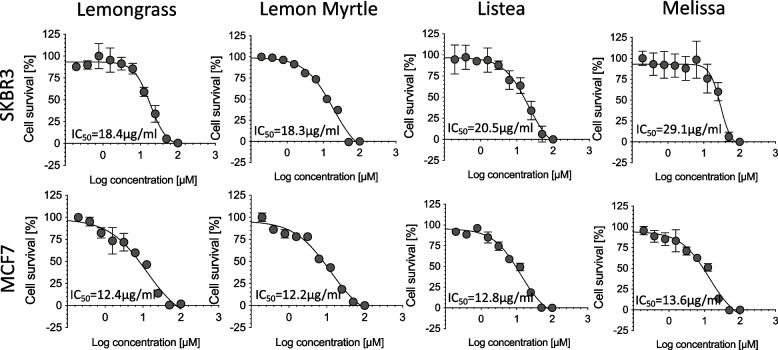
Antitumor effect of each essential oil. Each type of breast cancer cells (SKBR3 and MCF7) was incubated with varying doses of four essential oils for 48 h. The MTT assay was performed and the IC_50_ was analyzed. Each bar represents the mean ± SD of triplicate experiments

The MTT assay examined the tumor growth inhibitory effects of these four essential oils when acting directly on breast cancer cells. The IC_50_ for the SKBR3 cells was the lowest as 18.3 µg/mL for lemon myrtle and the highest as 29.1 µg/mL for the Melissa essential oil (Fig. 2). The IC_50_ values for the MCF7 cells was also shown the lowest as 12.2 µg/mL for Lemon Myrtle. Thus, we concluded that all four essential oils inhibited the proliferation of the breast cancer cells and Lemon Myrtle had the strongest activity when applied directly to the cancer cells.

### Cell morphology changes due to the volatile components of essential oil

**Fig. 3:**
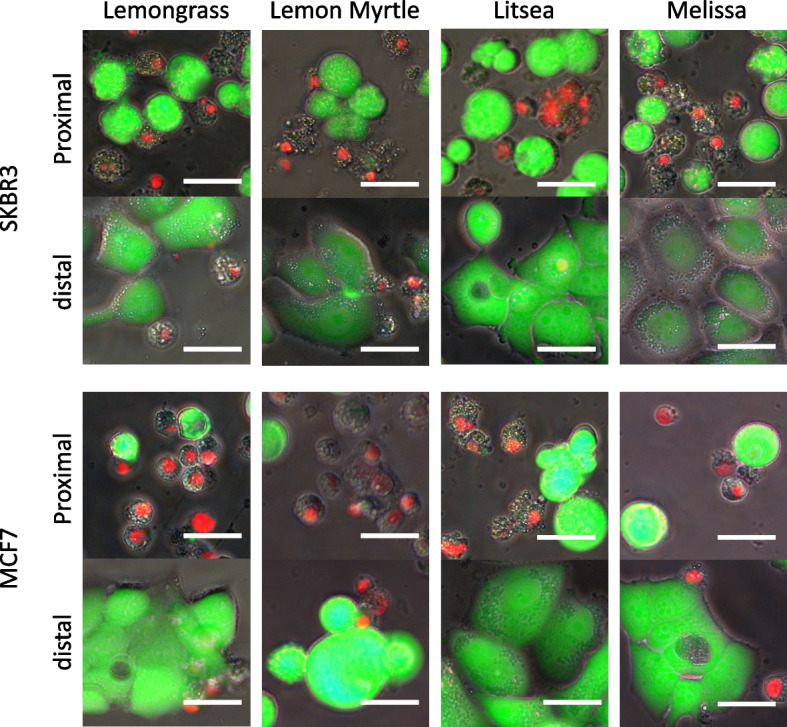
Morphological changes in breast cancer cells due to volatile components of essential oils. Two types of breast cancer cells (SKBR3 and MCF7) were incubated with four 500-fold diluted essential oils for 48 h. Morphological changes in breast cancer cells were analyzed by fluorescence microscopy (400x) after staining with Calcein-AM (Cal) and propidium iodide (PI). Phase contrast modes (PC) and merged modes (Mer) are also shown. The white bar equals 20 μm

The cellular changes in the volatile components of the four essential oils were also compared. Cancer cells in wells near of the 500-fold diluted essential oil (proximal group) and cancer cells in the wells farthest from that essential oil (distal group) were stained with calcein-AM and PI and analyzed using a fluorescence microscope (Fig. 3). In both cell lines, many red-stained dead cell nuclei were observed in the proximal group, whereas a lot of green-stained viable cells were observed in the distal group. The ratio of dead cells to living cells in the proximal group was slightly lower in Melissa essential oil, but no clear difference was observed in Lemongrass, Lemon Myrtle, or Litsea. Although the number of dead cells was smaller than that of the proximal group, some cancer cells in the distal group also showed cell death. The ratio of dead cells and viable cells in the distal group was almost the same for each essential oil.

### Antitumor effect by transpiration component of each essential oil

**Fig. 4:**
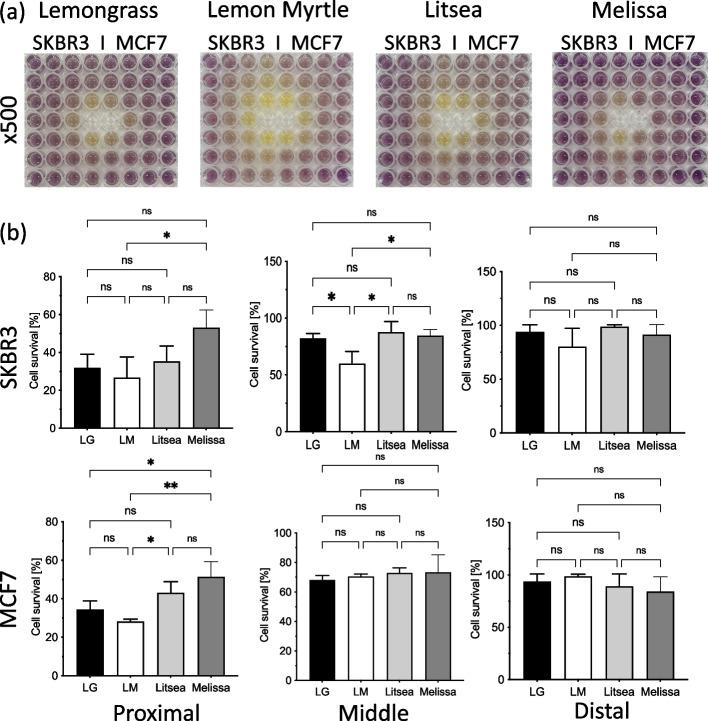
Antitumor effect by transpiration component of each essential oil. **a** Two types of breast cancer cells (SKBR3 and MCF7) were incubated in a 96-well plate, and four types of essential oils were transferred into the two center wells (LG: lemongrass, LM: lemon myrtle, litsea, and melissa). After 48 h, MTT assay was performed, and the results for the plate are shown. **b** The average of intensities of each group (proximal, middle, and distal) is shown in the bar graph. Each bar represents the mean ± SD of triplicate experiments (ns: not significant, *: *p* < 0.05 and **: *p* < 0.01)

The MTT assay was used with each of the four essential oils diluted 500-fold and the changes in cell growth inhibitory effects depending on the distances from the center were compared (Fig. 4a). Lemon myrtle suppressed the proliferation of the breast cancer cells the most and melissa suppressed cell proliferation the least. A plate reader was used to measure the absorbance of each well and the cell survival rate (%) of the three groups were compared based on their distances to the essential oil: proximal group (0.8–1.0 cm), middle group (1.6–2.0 cm), and distal group (2.4–3.0 cm) (Fig. 4b). In the proximal group, both cell lines were significantly suppressed by Lemon Myrtle (*p* < 0.05), whereas Lemongrass and Litsea displayed similar growth rates, and Melissa had the weakest effect on the proliferation rates of the breast cancer cells. Lemon Myrtle also significantly decreased the proliferation rate of SKBR3 in the middle group, but no clear difference was observed in the MCF7 cells. No differences were observed in the growth inhibitory effects of each essential oil against SKBR3 and MCF7 in the distal group.

### Effect of volatile components of each essential oil on normal cells

**Fig. 5:**
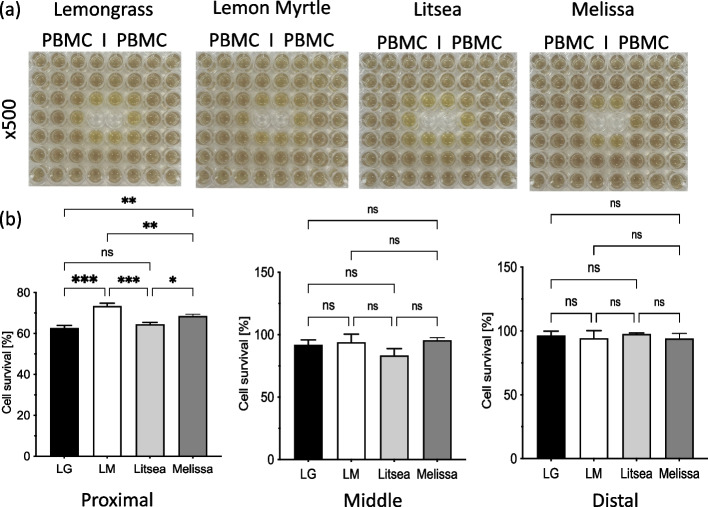
Effect of volatile components of each essential oil on healthy cells. **a** PBMCs were incubated in a 96-well plate, and four types of essential oils were transferred into the two center wells (LG: lemongrass, LM: lemon myrtle, litsea, and melissa). After 48 h, the MTT assay was performed, and the results for the plate are shown. **b** The average of the intensities of each group (proximal, middle, and distal) are shown in the bar graph. Each bar represents the mean ± SD of triplicate experiments (ns: not significant, *: *p* < 0.05, **: *p* < 0.01, ***: *p* < 0.005)

The MTT assay was also used to compare the effects of the volatile components of these essential oils on normal human PBMC (Fig. 5). In the proximal group, lemon myrtle did not suppress cell proliferation more significantly than the other essential oils. No significant differences were observed between the essential oils in the middle group and the proximal group, and cytotoxicity to the PBMC was very low. These results indicate that lemon myrtle does not affect normal cells, even though the two breast cancer cell lines studied here experienced strong cytostatic effects at the same dose.

### Comparative study of constituents in each essential oil

To clarify why lemon myrtle exhibited the strongest antitumor effects, GC–MS was used to analyze the components of each essential oil. The components whose content in the essential oil was 0.1% or more are listed in Table 2.

**Table 2:** Components and concentrations (%) in each essential oil

		Lemon grass	Lemon Myrtle	Litsea	Melissa	Chemical Class
1	Geranial	40.23	44	39.47	23.18	Monoterpene Aldehyde
2	beta-Caryophyllene	1.54	0.16	1.02	17.61	Sesquiterpene Hydrocarbon
3	Neral	29.81	39.77	29.05	17.16	Monoterpene Aldehyde
4	Limonene	0.33	/	14.37	0.53	Monoterpene Hydrocarbon
5	Germacrene D	0.24	0.02	0.01	9.63	Sesquiterpene Hydrocarbon
6	Geraniol	8	2.08	0.63	2.99	Monoterpene Alcohol
7	Geranyl acetate	5.35	/	0.03	2.23	Monoterpene Ester
8	trans-Isocitral	1.33	3.9	1.96	0.86	Monoterpene Aldehyde
9	Citronellal	0.25	0.24	0.62	3.38	Monoterpene Aldehyde
10	trans-beta-Ocimene	0.19	0.08	0.01	2.7	Monoterpene Hydrocarbon
11	cis-Isocitral	0.25	2.27	1	/	Monoterpene Aldehyde
12	6-Methyl-5-hepten-2-one	1.67	1.62	0.82	2.06	Aliphatic Ketone
13	Sabinene	/	/	1.81	/	Monoterpene Hydrocarbon
14	Myrcene	0.08	0.29	1.51	0.16	Monoterpene Hydrocarbon
15	alpha-Pinene	0.23	/	1.46	/	Monoterpene Hydrocarbon
16	delta-Cadinene	0.38	0.04	0.04	1.44	Sesquiterpene Hydrocarbon
17	2-Nonanone	1.38	/	/	/	Aliphatic Ketone
18	gamma-Cadinene	1.29	/	/	0.5	Sesquiterpene hydrocarbon
19	alpha-Humulene	0.18	0.02	0.1	1.28	Sesquiterpene Hydrocarbon
20	Nerol	/	1.29	0.33	1.05	Monoterpene Alcohol
21	Linalool	1.14	0.68	0.98	1	Monoterpene Alcohol
22	Camphene	1.12	/	0.27	/	Monoterpene Hydrocarbon
23	1,8-Cineole	0.1	/	1.09	/	Monoterpene Ether
24	beta-Pinene	0.02	/	1.08	/	Monoterpene Hydrocarbon
25	Caryophyllene oxide	0.56	/	0.13	1.04	Sesquiterpene Ether

Geranial was the most abundant component in the four essential oils, followed by neral. The resulting total citral levels were 70.04% in lemongrass, 83.77% in lemon myrtle, 78.52% in litsea, and 40.34% in melissa. The citral content was highest in lemon myrtle, which was consistent with the results of the antitumor effects (Fig. 4). Thus, we determined that citral was the most abundant tumor growth inhibitor in these essential oils.

### Comparison of citral concentrations in volatile components of essential oils

**Fig. 6:**
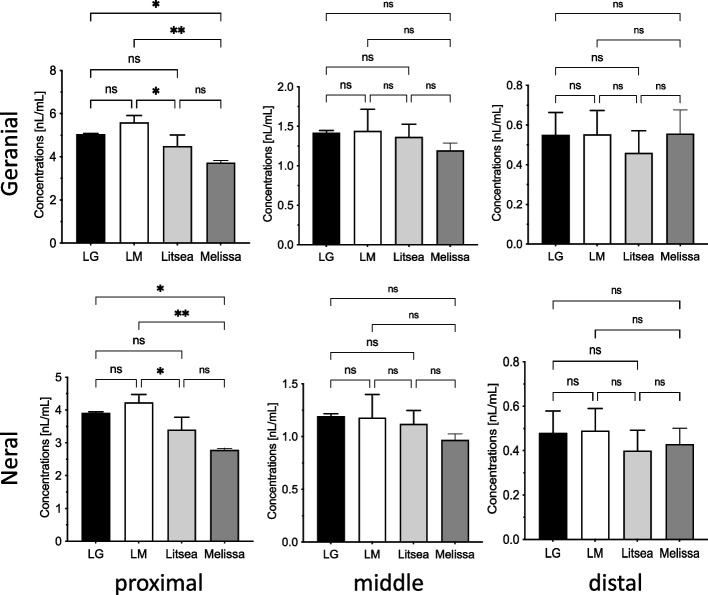
Citral concentrations in the volatile components of essential oils. The distance from the essential oil [proximal group (0.8–1.0 cm), middle group (1.6–2.0 cm), and distal group (2.4–3.0 cm)] and the concentration of volatile components (Geranial, Neral) in each oil (LG: lemongrass, LM: lemon myrtle, litsea, and melissa) was compared by GC–MS analysis. Each bar represents the mean ± SD of triplicate experiments (ns: not significant, *: *p* < 0.05, **: *p* < 0.01, ***: *p* < 0.005)

The citral adsorption rate of the MonoTrap RCC18 placed in each well was quantified using gas chromatography (Fig. 6). In the proximal group, the average geranial and neral concentrations were 5.0 ul/ml and 3.5 ul/ml, respectively. The concentrations were significantly higher in lemon myrtle than in the other essential oils. No significant differences in citral concentrations were found in the middle and distal group essential oils. The citral concentrations (IC_50_) at which each essential oil showed cytotoxicity to breast cancer cells was 1.67 ul/ml for geranial and 1.31 ul/ml for neral (Figs. 4 and 6).

### Citral antitumor effects

**Fig. 7:**
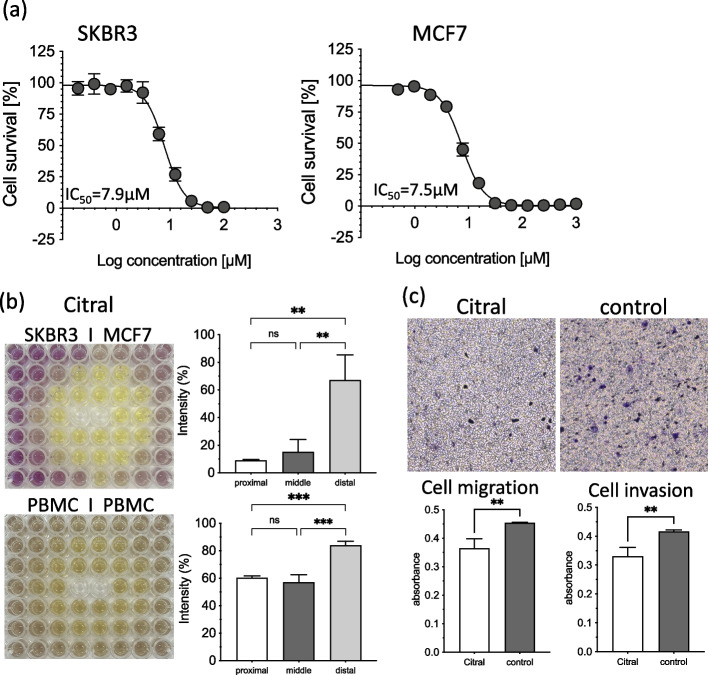
Antitumor effects of citral. **a** Each type of breast cancer cells (SKBR3 and MCF7) was incubated with varying dose of citrals for 48 h. The MTT assay was performed and the IC_50_ was determined. Each bar represents the mean ± SD of triplicate experiments. **b** Cancer cells (SKBR and MCF7) or PBMCs were incubated in a 96-well plate, and 500-fold diluted citral was transferred into the two center wells. After 48 h, the MTT assay was performed, and the results for the plate are shown. The average of the intensities of each group (proximal, middle, and distal) are presented in the bar graph. Each bar represents the mean ± SD of triplicate experiments (ns: not significant, *: *p* < 0.05, **: *p* < 0.01 and ***: *p* < 0.005). **c** The cell migration and cell invasion assays were performed on SKBR3 breast cancer cells exposed to the volatile components in citral at 500-fold dilution. The results of the invasion assay are shown. The average absorbances are presented in the bar graph

We investigated the antitumor effect of citral in vitro. When citral was directly applied to breast cancer cells, the IC_50_ was 7.9 uM for SKBR3 and 7.5 uM for MCF7 (Fig. 7a). When the volatile components of citral acted on the breast cancer cells, cell proliferation in the proximal to partially distal groups was suppressed at 500-fold dilution (Fig. 7b). Intensity decreased to 9.0% in the proximal group, 15.2% in the middle group, and 67.1% in the distal group; thus, showing a stronger antitumor effect than in lemon myrtle. When normal PBMC reacted with citral, the intensity was 60.3% in the proximal group, 57.1% in the middle group, and 83.9% in the distal group. The cell migration and cell invasion assays were performed on SKBR3 breast cancer cells exposed to the volatile components in citral at 500-fold dilution (Fig. 7c). The ability of the SKBR3 cell line to migrate and invade was significantly reduced by the volatile components of citral (*p* < 0.01). Thus, transpired citral has a strong antitumor effect.

## Discussion

### Diagnosis and treatment of breast cancer

Aromatherapy for the prevention and treatment of cancer has been practiced as an alternative therapy using plant-derived essential oils [10]. For example, it has been shown that frankincense induces apoptosis in breast cancer cells and that lavender induces apoptosis in stomach, skin, and breast cancer cells [11, 12]. Carvacrol is a monoterpene extracted from, for example, *Thymus vulgaris* (thyme) and *Origanum vulgale* (oregano) and kills multiple cancer cells such as breast, stomach, colorectal, and liver cancer by arresting the cell cycle [13, 14]. In the present study, lemongrass, lemon myrtle, litsea, and melissa had the strongest antitumor effects (Fig. 1) on breast cancer cells. In contrast to other studies that have shown that each essential oil directly acts on cancer cells, this study compares the antitumor effects of the transpiration components of each essential oil. No previous reports have focused on the transpiration components of each essential oil and compared them. This method has good value as a basic study in the potential treatment of cancer with essential oils.

### Lemon myrtle

Lemon Myrtle (*Backhousia citriodora*), a shrub of the Myrtaceae family, is a species native to the rainforests of the Queensland coastal region of Australia [15]. It has a rich lemon scent, contains citral, and is used as a traditional spice in Australia. Previous studies have shown that lemon myrtle has high antioxidant and anti-inflammatory effects due to its suppression of the production of inflammatory mediators, such as nitric oxide (NO) [16–18]. In addition, lemon myrtle has been found to act directly on colon, stomach, and bladder cancer cells by inducing apoptosis via Caspase3 [19]. In this study, the transpiration component of lemon myrtle essential oil displayed strong cytotoxicity against breast cancer cells but did not display any toxicity in normal white blood cells. Lemon myrtle's carcinogenic effect was the strongest of the four tested essential oils, which might be due to it having the highest citral concentration (a mixture of neral and geranial isomers) of the four oils. Citral is a monoterpene and is known to be a component with a low molecular weight and high volatility. These results suggest that lemon myrtle may be beneficial as a cancer treatment due to its antitumor effects.

### Lemongrass

Lemongrass (*Cymbopogon* spp.) is a perennial plant of the grass family and is widely used in tropical countries, especially in Southeast Asia. The lemongrass genus includes about 180 species, such as *Cymbopogon citratus* and *Cymbopogon flexuosus*. Lemongrass essential oil contains numerous bioactive compounds, including citral, citronellol, citronellal, limonene, linalool, and nerol [20]. Lemongrass essential oil has been reported to display antibacterial, anti-inflammatory, anticancer, antimutagenic, and antidiabetic activities [21]. In particular, *C. citratus* essential oil has been reported to exert potent cytotoxic activity against various cancer cell lines [22, 23].

#### Melissa

Melissa (*Melissa officinalis* L.) is a perennial plant of the Labiatae family, also known as lemon balm [24]. It generally grows in the Mediterranean region and western Asia and is intensively cultivated in Europe. Melissa has various medicinal effects, such as hypoglycemic, hepatoprotective, antibacterial, antidepressant, hypnotic, sedative, and antioxidant effects [25]. Melissa has also been reported to exhibit cytotoxic effects on lung, breast, ovarian, pancreatic, colon and brain tumor cells [26, 27]. Rosmarinic acid in melissa is reported to display a strong antitumor effect [28].

### Litsea

Listea (*Litsea cubeba*) is a 3–10 m evergreen tree or shrub widely distributed in Southeast Asia, southern China, Japan, and Taiwan and is used as a traditional medicine to treat various ailments [29]. Litsea exhibits cytotoxicity against lung, breast, gastric, uterine, ovarian, and hepatoma cells [30, 31] and potentiates the cytotoxicity of oxaliplatin [32]. Seal et al. reported that the volatile components of litsea essential oil inactivate the protein kinase Akt in lung cancer cells, induce apoptosis through mitochondrial membrane potential depolarization, and arrest the cell cycle in the G1/S phase, thereby inhibiting cancer cell growth [33]. Since the volatile components of litsea essential oil can be delivered directly to the lung tissue by inhalation, it is expected that a new treatment method will be established.

### Citral

Citral (3,7-dimethyl-2,6-octadien-1-al) is an aldehyde component found in essential oils extracted from citrus, lemongrass, and ginger [34]. Citral induces apoptosis in gastric, colon, breast, uterine, ovarian, and prostate cancer cells [35–38]. Citral also downregulates aldehyde dehydrogenase (ALDH) activity and Wnt signaling, thereby suppressing spheroid formation, one of the mechanisms of drug resistance acquisition [39, 40]. Cravotto et al. reported that the oral administration of citral to mice and rats resulted in its rapid absorption in the gastrointestinal tract [41]. Although most of the citral applied to the skin was volatilized and lost, the remaining citral was absorbed into the body. Citral in the blood was rapidly metabolized and excreted in the urine. Nanostructured lipid carriers encapsulating citral (NLC-Citral) were formed to prevent citral loss by volatilization and promote its uptake into tumor cells, thus suppressing breast cancer cell proliferation and metastasis in vivo and in vitro [42, 43].

Oral or dermal lethal dose (LD50) values of citral in rats exceeded 1000 mg/kg, with the acute toxicity of citral reported to be low in rodents [41]. In this study, each transpiration component of the four essential oils displayed almost no cytotoxicity to normal cells (PBMC) in the middle and distal groups (Fig. 5). Lemon Myrtle, which had the highest citral content, was significantly less cytotoxic than the other three essential oils; however, when citral alone was reacted with PBMC, it showed relatively strong cytotoxic effects to the cells in the middle group, which was equivalent to that of the proximal group (Fig. 7b). Thus, citral-rich lemon myrtle may contain a component that reduces citral’s cytotoxicity to normal cells. Further research is needed to identify this component. The results of this study indicate that essential oils that contain a large amount of citral, such as lemon myrtle, could be promising in future cancer treatments due to their transpiration components.

This study found that the volatile components of essential oils that contain a large amount of citral, such as Lemongrass, Lemon Myrtle, and Litsea, all have high antitumor activity. Furthermore, it was shown that the higher the citral concentration in the volatile fraction, the stronger the antitumor activity. These essential oils, along with Litsea, are considered important essential oils for new treatments by inhaling volatiles.

## Conclusion

The volatile components of lemongrass, lemon myrtle, litsea, and melissa essential oils were shown to have high antitumor activities, with lemon myrtle displaying the strongest activity. Moreover, these four essential oils displayed a low cytotoxicity to normal PBMCs. All of these essential oils contained a large amount of citral, which is thought to induce cell death in breast cancer cells in its volatilized state. When citral was reacted independently with breast cancer cells, it showed a strong antitumor effect that reduced cell proliferation, migration, and invasiveness. Based on these findings, the four essential oils could be used to induce cell death in breast cancer cells via the transpiration of citral; thus, suggesting the possibility of its use as an effective ingredient for cancer treatment.

## Data Availability

The datasets generated and/or analyzed during the current study available from the corresponding author on reasonable request.

## References

[1] Cancer statistics and graph database. Center for the cancer control and information services. 2021. Available from: http//www.ganjoho.jp/public/statistics/backnumber/2021_jp.html.

[2] Shimoi T, Nagai S, Yoshinami T, Takahashi M, Arioka H (2020). The Japanese breast cancer society clinical practice guidelines for systemic treatment of breast cancer, 2018 edition. Breast Cancer.

[3] Rosenkaimer S, Sieburg T, Winter L, Mavratzas A, Hoffman WK (2022). Adverse cardiovascular effects of anti-tumor therapies in patients with breast cancer: A single-center cross-sectional analysis. Anticancer Res.

[4] Christianson DW (2017). Structural and chemical biology of terpenoid cyclases. Chem Rev.

[5] Salakhutdinov NF, Volcho KP, Yarovaya OI (2017). Monoterpenes as a renewable source of biologically active compounds. Pure Appl Chem.

[6] Viktorová J, Stupák M, Rˇehorˇová K, Dobiasová S, Hoang L, Hajšlová J, Thanh TV, Tri LV, Tuan NV, Ruml T (2020). Lemongrass essential oil does not modulate cancer cells multidrug resistance by citral - Its dominant and strongly antimicrobial compound. Foods.

[7] Jeong LH, Sang JH, Joong KD, Hee NY, Yeon YD, Hong JT (2008). Inhibitory effect of citral on NO production by suppression of iNOS expression and NF-kappa B activation in RAW264.7 cells. Arch Pharm Res.

[8] Sobral MV, Xavier AL, Lima TC, De Sousa DP (2014). Antitumor activity of monoterpenes found in essential oils. Sci World J.

[9] Zielińska-Błajet M, Pietrusiak P, Feder-Kubis J (2021). Selected monocyclic monoterpenes and their derivatives as effective anticancer therapeutic agents. Int J Mol Sci.

[10] Bayala B, Bassole I, Scifo R, Gnoula C, Morel L, Lobaccaro JM, Simper J (2014). Anticancer activity of essential oils and their chemical components - a review. Am J Cancer Res.

[11] Chen Y, Zhou C, Ge Z, Liu Y, Liu Y, Feng W, Li S, Chen G, Wei T (2013). Composition and potential anticancer activities of essential oils obtained from myrrh and frankincense. Oncol Lett.

[12] Boukhatem MN, Sudha T, Darwish NHE, Chader H, Belkadi A, Rajabi M, Houche A, Benkebailli F, Oudjida F, Mousa SA. A new eucalyptol-rich Lavender (Lavandula stoechas L.) essential oil: emerging potential for therapy against inflammation and cancer. Molecules. 2020;25(16):3671. 10.3390/molecules25163671. 10.3390/molecules25163671 PMC7463424 32806608

[13] Jaafari A, Tilaoui M, Mouse HA (2012). Comparative study of the antitumor effect of natural monoterpenes: relationship to cell cycle analysis. Brazilian Journal of Pharmacognosy.

[14] Yin QH, Yan FX, Zu XY, Wu YH, Wu XP, Liao MC, Deng SW, Yin LL, Zhuang YZ (2012). Anti-proliferative and pro-apoptotic effect of carvacrol on human hepatocellular carcinoma cell line HepG-2. Cytotechnology.

[15] Shin S-Y, Kim J-H, Kho K-H, Lee M (2020). Anti-inflammatory and anti-oxidative activities of lemon myrtle (Backhousia citriodora) leaf extract. Toxicol Rep.

[16] Kang E-J, Lee J-K, Park H-R, Kim H, Kim H-S, Park J (2020). Antioxidant and anti-inflammatory activities of phenolic compounds extracted from lemon myrtle (Backhousia citriodora) leaves at various extraction conditions. Food Sci Biotechnol.

[17] Emmanuel Janaka Rochana Rupesinghe, Andrew Jones, Ross Andrew Shalliker, Sercan Pravadali-Cekic. A rapid screening análisis of antioxidant compounds in native australian food plants using multiplexed detection with active flow technology columns. Molecules. 2016,21,118. 10.3990/molecules21010118. 10.3390/molecules21010118 PMC6273279 26805792

[18] Guo Yu, Sakulnarmrat K, Konczak I (2014). Anti-inflammatory potential of native Australian herbs polyphenols. Toxicol Rep.

[19] Sakulnarmrat K, Fenech M, Thomas P, Konczak I (2013). Cytoprotective and pro-apoptotic activities of native Australian herbs polyphenolic-rich extracts. Food Chem.

[20] Viktorová J, Stupák M, Řehořová K, Dobiasová S, Hoang L, Hajšlová J, Thanh TV, Tri LV, Tuan NV, Ruml T (2020). Lemongrass essential oil does not modulate cancer cells multidrug resistance by Citral-Its dominant and strongly antimicrobial compound. Foods.

[21] Mukarram M, Choudhary S, Khan MA, Poltronieri P, Khan MMA, Ali J, Kurjak D, Shahid M (2021). Lemongrass essential oil components with antimicrovial and anticancer activites. Antioxidants (Basel).

[22] Van HTK, Quy NM, Ha DTV (2018). Chemical composition and cytotoxic activity of the essential oils of Cymbopogon citratus L. grown in Phu Tho province. Vietnam Journal of Biotechnology.

[23] Trang DT, Hoang TKV, Nguyen TTM, Van Cuong P, Dang NH, Dang HD, Nguyen Quang T, Dat NT (2020). Essential oils of Lemongrass (Cymbopogon citratus stapf) induces apoptosis and cell cycle arrest in A549 lung cancer cells. Biomed Res Int.

[24] Miraj S, Rafieian-Kopaei, Kiani S. Melissa officinalis L: A review study with an antioxidant prospective. J Evid Based Complementary Altern Med. 2017;22(3):385–394. 10.1177/2156587216663433. 10.1177/2156587216663433 PMC5871149 27620926

[25] Araj-Khodaei M, Noorbala AA, Yarani R, Emadi F, Emaratkar E, Faghihzadeh S, Parsian Z, Alijaniha F, Kamalinejad M, Naseri M (2020). A comparative study of Melissa officinalis leaves and stems ethanolic extracts in terms of antioxidant, cytotoxic, and antiproliferative potential. BMC Complement Med Ther.

[26] Ramanauskiene K, Raudonis R, Majiene D (2016). Rosemarinic acid and Melissa officinalis extracts differently affect glioblastoma cells. Oxid Med Cell Longev.

[27] Mouhid L, Gómez de Cedrón M, Vargas T, García-Carrascosa E, Herranz N, García-Risco M, Reglero G, Fornari T, Ramírez de Molina A. Identification of antitumoral agents against human pancreatic cancer from Asteraceae and Lamiaceae plant extracts. BMC Complement Altern Med. 2018;18(1):254. 10.1186/s12906-018-2322-6. 10.1186/s12906-018-2322-6 PMC6142333 30223811

[28] Encalada MA, Hoyos KM, Rehecho S, Berasategi I, de Ciriano MG, Ansorena D, Astiasarán I, Navarro-Blasco I, Cavero RY, Calvo MI (2011). Anti-proliferative effect of Melissa officinalis on human cancer cell line. Plant Foods Hum Nutr.

[29] Kong DG, Zhao Y, Li GH, Chen BJ, Wang XN, Zhou HL, Lou HX, Ren DM, Shen T (2015). The genus Litsea in traditional Chinese medicine: an ethnomedical, phytochemical and pharmacological review. J Ethnopharmacol.

[30] Su YC, Hsu KP, Wang EI, Ho CL (2013). Compositions and in vitro anticancer activities of the leaf and fruit oils of Litsea cubeba from Taiwan. Nat Prod Commun.

[31] Zhang W, Hu JF, Lv WW, Zhao QC, Shi GB (2012). Antibacterial, antifungal and cytotoxic isoquinolinealkaloids from Litsea cubeba. Molecules.

[32] Yu BB, Dong SY, Yu ML, Jiang GJ, Ji J, Tong XH (2014). Total flavonoids of Litsea Coreana enhance the cytotoxity of oxaliplatin by Increasing Gap junction intercellular communication. Biol Pharm Bull.

[33] Seal S, Chatterjee P, Bhattacharya S, Pal D, Dasgupta S, Kundu R, Mukherjee S, Bhattacharya S, Bhuyan M, Bhattacharyya PR, Baishya G, Barua NC, Baruah PK, Rao PG, Bhattacharya. Vapor of volatile oils fromLitsea cubeba seed induces apoptosis and causes cycle arrest in lung cancer cells. PLoS One. 2012;7(10):e47014. 10.1371/journal.pone.0047014. 10.1371/journal.pone.0047014 PMC3473030 23091605

[34] Nigjeh SE, Yeap SK, Nordin N, Rahman H, Rosli R (2019). In vivo antitumor effects of Citral on 4T1 breast cancer cells via induction of apoptosis and downregulation of aldehyde dehydrogenase activity. Molecules.

[35] Balusamy SR, Ramani S, Natarajan S, Kim YJ, Perumalsamy H (2019). Integrated transcriptome and in vitro analysis revealed anti-proliferative effect of citral in humnstomach cancer through apoptosis. Sci Rep.

[36] Balusamy SR, Perumalsamy H, Veerappan K, Huq MA, Rajeshkumar S, Lakshmi T, Kim YJ. Citral induced apoptosis through modulation of key genes involved in fatty acid biosynthesis in human prostate cancer cells: In silico and in vitro study. Biomed Res Int. 2020:6040727. 10.1155/2020/6040727. 10.1155/2020/6040727 PMC7103989 32258129

[37] Liu Y, Whelan RJ, Pattnaik BR, Ludwig K, Subudhi E, Rowland H, Claussen N, Zucker N, Uppal S, Kushner DM, Felder M, Patankar MS, Kapur A (2012). Terpenoids from Zingiber officinale (Ginger) induce apoptosis in endometrial cancer cells through the activation of p53. PLoS ONE.

[38] Sheikh BY, Sarker MMR, Kamarudin MNA, Mohan G (2017). Antiproliferative and apoptosis inducing effects of citral via p53 and ROS-induced mitochondrial-mediated apoptosis in human colorectal HCT115 and HT29 cell lines. Biomed Pharmacother.

[39] Nigjeh SE, Yeap SK, Nordin N, Kamalideghan B, Ky H, Rosli R (2018). Citral induced apoptosis in MDA-MB-231 spheroid cells. BMC Complement Altern Med.

[40] Thomas ML, de Antueno R, Coyle KM, Sultan M, Cruickshank BM, Giacomantonio MA, Giacomantonio CA, Duncan R, Marcato P (2016). Citral reduces breast tumor growth by inhibiting the cancer stem cell marker ALDH1A3. Mol Oncol.

[41] Cravotto G, Binello A, Baranelli E, Carraro P, Trotta F (2006). Cyclodextrins as food additives and in food processing. Curr Nutr Food Sci.

[42] Nordin N, Yeap SK, Rahman HS, Zamberi NR, Mohamad NE, Abu N, Masarudin MJ, Abdullah R, Alitheen NB (2020). Antitumor and anti-metastatic effects of Citral-loaded nanostructured lipid carrier in 4T1-induced breast cancer mouse model. Molecules.

[43] Nordin N, Yeap SK, Rahman HS, Zamberi NR, Abu N, Mohamad NE, How CW, Masarudin MJ, Abdullah R, Alitheen NB (2019). In vitro cytotoxicity and anticancer effects of citral nanostructured lipid carrier on MDA MBA-231 human breast cancer cells. Sci Rep.

